# Hiding in plain sight: a partial deletion of BRCA1 exon 7 undetectable by MLPA is a Nepali founder variant

**DOI:** 10.1136/jmg-2024-110422

**Published:** 2024-12-11

**Authors:** Virginia Clowes, Jenny C Taylor, Alistair T Pagnamenta

**Affiliations:** 1 North West Thames Regional Genetics Service, Northwick Park Hospital, London, UK; 2 Centre for Paediatrics and Child Health, Faculty of Medicine, Imperial College London, London, UK; 3 Oxford NIHR Biomedical Research Centre, Centre for Human Genetics, University of Oxford, Oxford, UK; 4 Institute of Biomedical and Clinical Science, University of Exeter Medical School, Royal Devon University Healthcare NHS Foundation Trust, Exeter, Devon, UK

**Keywords:** Founder Effect, Genetic Testing, Genomics, Sequence Deletion, Medical Oncology

 Multiplex ligation-dependent probe amplification (MLPA) has been used diagnostically for the *BRCA1/BRCA2* genes for >20 years and deletions/duplications involving *BRCA1* are known to account for a substantial fraction of diagnostic alleles.^1^ We describe a family from 100 000 Genomes Project (100kGP) with a significant family history of breast/ovarian cancer harbouring a 178 bp *BRCA1* deletion. Due to its location, the deletion was cryptic to both next-generation sequencing (NGS) gene panel and MLPA testing. Further interrogation of variant databases suggested it to be a Nepali founder variant and highlights the potential for nearby variants on the disease haplotype to lead to inconsistent Human Genome Variation Society (HGVS) nomenclature.

The proband (now deceased) was diagnosed with ovarian cancer and her affected first cousin was diagnosed with first breast, then ovarian cancer. The family is of Nepali ancestry and there is a positive family history, with three females in the previous generation diagnosed with early-onset breast cancer. Ages at diagnosis were 41–50 years (online supplemental figure S1). Previous genetic testing included the SALSA MLPA Probemix BRCA1-P002-C1 (MRC-Holland) and NGS with the Roche 454 GS-FLX platform, with a minimum 30× coverage. These tests were performed in 2011 and did not identify any plausible diagnostic variants. The proband and her cousin were therefore recruited to the 100kGP in mid-2016.

The 100kGP is a UK-wide study that helped demonstrate the utility of genome sequencing (GS) for patients with a range of rare disease and cancer.^2^ Library preparation used the TruSeq PCR-free high-throughput kit and DNA from EDTA blood tubes. GS was performed on an Illumina HiSeqX, with 150 bp paired-end reads. Alignment and variant calling used the North Star Version 4 Workflow (NSV4, V.2.6.53.23) and the GRCh38 reference. GS for the proband and her affected cousin generated a mean coverage of 40× and 52×, respectively. Sequencing and bioinformatic preprocessing was conducted centrally by Genomics England. The primary analysis, performed in mid-2019, focused on 10 genes linked to familial breast cancer (PanelApp V.1.13: *ATM*, *BRCA1*, *BRCA2*, *CHEK2*, *PALB2*, *PTEN*, *RAD51C*, *RAD51D*, *STK11*, *TP53*), but no likely-pathogenic variants were identified.

Detection of structural variants (SVs) used Manta and Canvas algorithms.^3 4^ In this study, combined SV calls were prioritised using allele frequencies determined from aggregated data for 71 408 individuals.^5^ Read alignments were viewed using IGV v2.15.4. A 178 bp deletion in *BRCA1* (NC_000017.11:g.43099608_43099786del) was called by Manta that was shared by both affected individuals but not identified in any other 100kGP participants. Due to the size of the deletion, Canvas did not pick up any CNVs at this locus and a 4.2 Mb CanvasREF call (chr17:41,276,119–45,511,623) spanned the entirety of *BRCA1*. The deletion only removes 12 bp of exon 7 and thus does not impact the nearest MLPA probe binding site (figure 1). Together with the normal result from Canvas, this explains why the deletion had previously remained undetected. Review of probe designs for the latest iterations of *BRCA1* MLPA panels suggests this deletion would also be cryptic to P087 and other newer kits (Lillit Atanesyan, personal communication, 2024). Haplotype analysis (online supplemental table S1, figure S2) identified a single nearby coding SNV, NM_005899.5(NBR1):c.1244T>C, p.Met415Thr, which is also private to this family across the 100kGP and which could potentially be used as a tagging SNV.

**Figure 1:**
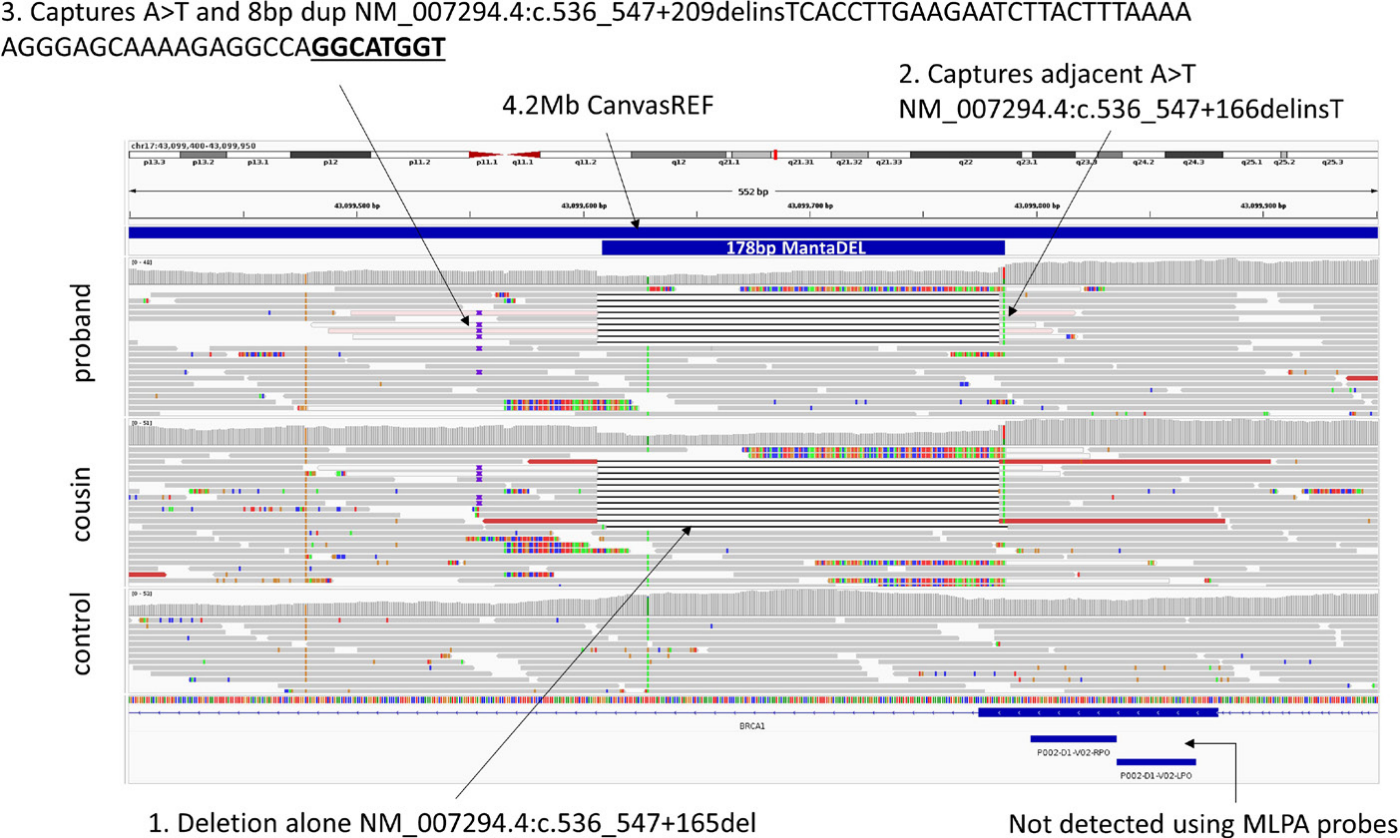
Read alignments supporting partial exon deletion in *BRCA1* shared by affected cousins with breast and ovarian cancer. Due to the presence of two nearby variants *in cis* with the deletion, there are three options for Human Genome Variation Society annotation, as indicated. Due to how the reads are aligned, the T insertion appears as a A>T substitution adjacent to the deletion in IGV. Region shown is chr17:43,099,400–43,099,950 (GRCh38). Reads are visualised using the ‘show soft-clipped bases’ and ‘collapsed’ settings within IGV, with reads coloured by ‘insert-size and pair orientation’. Positions of binding sites for exon 7 MLPA probes (MRC Holland kit P002) are immediately distal to the deletion. The control (bottom track) is a randomly selected sample from the same sequencing batch as the proband.

To facilitate optimal PCR primer design, surrounding genomic features were assessed and the ClinVar CNV track was loaded into our UCSC genome browser session (https://genome.ucsc.edu/s/AlistairP/BRCA1_del_final). Review of ClinVar CNVs in this region identified three similar deletions submitted between 2019 and 2023 (table 1). Although the three deletions appeared to share an identical distal breakpoint, the proximal end differed, and 2/3 annotations included 1 or 52 bp of inserted sequence. Scrutiny of these annotations, together with read alignments from the 100kGP, suggested that all three ClinVar entries represented an identical variant. This interpretation was substantiated through discussions with the submitting laboratories. Although all three HGVS annotations are correct, the ambiguity rests on whether the insertion of a T or a nearby variant found *in cis* (ie, present on the same disease-haplotype) is also captured. The NM_007294.4:c.536_547+166delinsT annotation includes the insertion of a T but appears as an adjacent SNV in IGV (figure 1). In contrast, c.536_547+209delinsTCACCTTGAAGAATCTTACTTTAAAAAGGGAGCAAAAGAGGCCAGGCATGGT captures both the SNV and a nearby 8 bp duplication. Although this information could be useful, for example, if designing PCR primers, a recent proposal to the HGVS committee (https://hgvs-nomenclature.org/stable/consultation/SVD-WG010/) recommends that ‘two variants that are separated by fewer than two intervening nucleotides should be described as a single ‘delins’ variant’. Therefore, the SV should be split into two variants, that is, c.[536_547+166delinsT;547+213_547+220dup].

**Table 1:** Founder deletion in *BRCA1* with 3 different annotations in ClinVar (www.ncbi.nlm.nih.gov/clinvar; data accessed 31 Jul 2024)

ClinVar variant ID/accession	531432	919622	2585827
ClinVar accession	VCV000531432.4	VCV000919622.8	VCV002585827.2
HGVS annotation used in ClinVar (NM_007294.4)	c.536_547+165del	c.536_547+166delinsT	c.536_547+209delinsTCACCTTGAAGAATCTTACTTTAAAAAGGGAGCAAAAGAGGCCAGGCATGGT
Number of submissions, classification	1 (Invitae), P	2 (Color Health, Invitae), LP/P	1 (Ambry Genetics), LP
Submission dates	September 2019	September 2019, May 2021	August 2023
Star rating	1	2	1
Ethnicity of families and other information provided by submitter	Tested individuals were of Nepalese descent. Variant detected in NGS data using split-reads. No confirmation method as MLPA probes do not overlap the deletion. However, NGS reads are of good enough quality to be confident that it is a true positive call (Labcorp Genetics, formerly Invitae, personal communication, 2023).	First submission relates to two clients, but neither provided ancestry information. Deletion identified with both Scalpel and an in-house developed split-read algorithm (Color Health, personal communication, 2023).For second submission, tested individuals were of Nepalese descent. Variant detected in NGS data using split-reads and validated using PacBio (Labcorp Genetics, formerly Invitae, personal communication, 2023).	The proband in this family was diagnosed with ovarian cancer in her early 50s and does appear to have Nepalese ancestry. Three additional family members were found to be carriers but have no personal history of cancer at the time of testing in their 30s to 40s. Picked up by NGS pipeline and then Sanger validated (Ambry Genetics, personal communication, 2024).Variant
Variant complexity captured by HGVS	–	NC_000017.11:g.43099786T>A*	NC_000017.11:g.43099786T>A* and NC_000017.11:g.43099558_43099565dup†
SpliceAI prediction‡	DL=0.99 (166), AL=0.92 (271)	DL=0.99 (167), AL=0.91 (272)	DL=0.99 (210), AL=0.93 (315)

All suggest exon skipping as a likely consequence.

* Private to this family in 100 kGP and not in gnomAD V.4.1.0. InsT appears as SNV at this position in IGV (figure 1) but could also be coded as NC_000017.11:g.43099609G>A.

† In vcf as 17:43099554A>AGCCACCAT. Private to this family in 100 kGP.

‡ Whether or not the nearby variants were taken into account did not significantly affect *in silico* predictions of splicing effect, as assessed with https://spliceailookup.broadinstitute.org.

ALacceptor lossDLdonor lossLPlikely pathogenicMLPAmultiplex ligation-dependent probe amplification NGSnext-generation sequencingPpathogenic

*In silico* analysis using SpliceAI^6 7^ predicted exon skipping and including the nearby variants in the input HGVS string had a limited effect on the score (table 1). Skipping of exon 7 (r.442_547del) would lead to a frameshift p.(Gln148Aspfs*51) and so the allele is likely to be functionally deleterious. The deletion-insertion was validated in an accredited laboratory and classified as pathogenic (PVS1 and PM2).

DNA sequencing and array technologies have shown rapid improvements in recent years.^8 9^ However, there remains a blind spot for variants that have a size range of 50–1000 bp, such as the deletion described here. These are too large to be identified by small variant calling algorithms but too small to be detected by arrays. To address this deficit, clinical testing laboratories often employ MLPA, whereby pairs of probes can be custom-designed to target around 40–50 loci. The 178 bp deletion described here highlights that partial exon deletions can remain undetected, even when MLPA probes lie in the same exon. With GS data, appropriate algorithms are required to detect deletions of this size. Following the generation of GS data, the variant took ~3.5 years to be uncovered, lengthening the family’s diagnostic odyssey and delaying opportunities for cascade screening in at-risk family members.

Interrogation of clinical variant databases suggests that the founder deletion is of Nepali origin (table 1). Although this deletion was not identified in a 2022 study on founder variants in the Nepalese population,^10^ the methods used in that study would be unlikely to identify this deletion, and there remained 31 families with unsolved breast cancer who could be screened. We anticipate that testing these and other families of this ethnicity using appropriate methodologies may identify further families with this cryptic variant.

Generally, if variable annotations are used for the same variant, replication of disease association may be missed and this can hinder variant prioritisation. In this particular case, we note that the star-rating system used in ClinVar could potentially result in the 178 bp deletion being filtered out by some analytical pipelines. Database submitters and curators should therefore pay close attention to overlapping structural variants reported previously when deciding whether nearby variants found *in cis* should be factored into the HGVS notation. Conversely, older submissions that use outdated HGVS notation should ideally be merged into newer records.

## supplementary material

10.1136/jmg-2024-110422 online supplemental file 1
