# Supplementary material for: Hiding in plain sight: a partial deletion of BRCA1 exon 7 undetectable by MLPA is a Nepali founder variant
Source: J Med Genet. 2024 Dec 11;62(2):e110422. doi: 10.1136/jmg-2024-110422 (PMC11877027; doi:10.1136/jmg-2024-110422)
Supplement: online supplemental file 1 [file jmg-62-2-s001.pdf]

Supplementary Information: Hiding in plain sight: a partial deletion of *BRCA1* exon 7 undetectable by MLPA is a Nepali founder variant

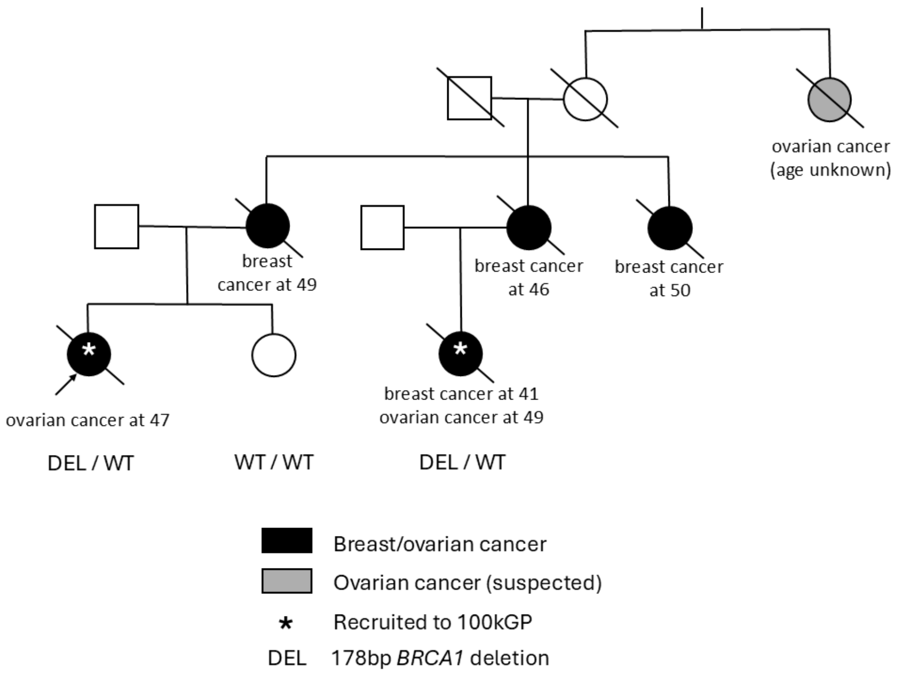

Figure S1: Simplified pedigree diagram of Nepali family with significant history of breast/ovarian cancer. The two individuals recruited to the 100k Genomes project were shown to harbour a heterozygous deletion overlapping exon 7 of *BRCA1*. Cascade testing of other at-risk family members to date has just included the proband's unaffected sister.

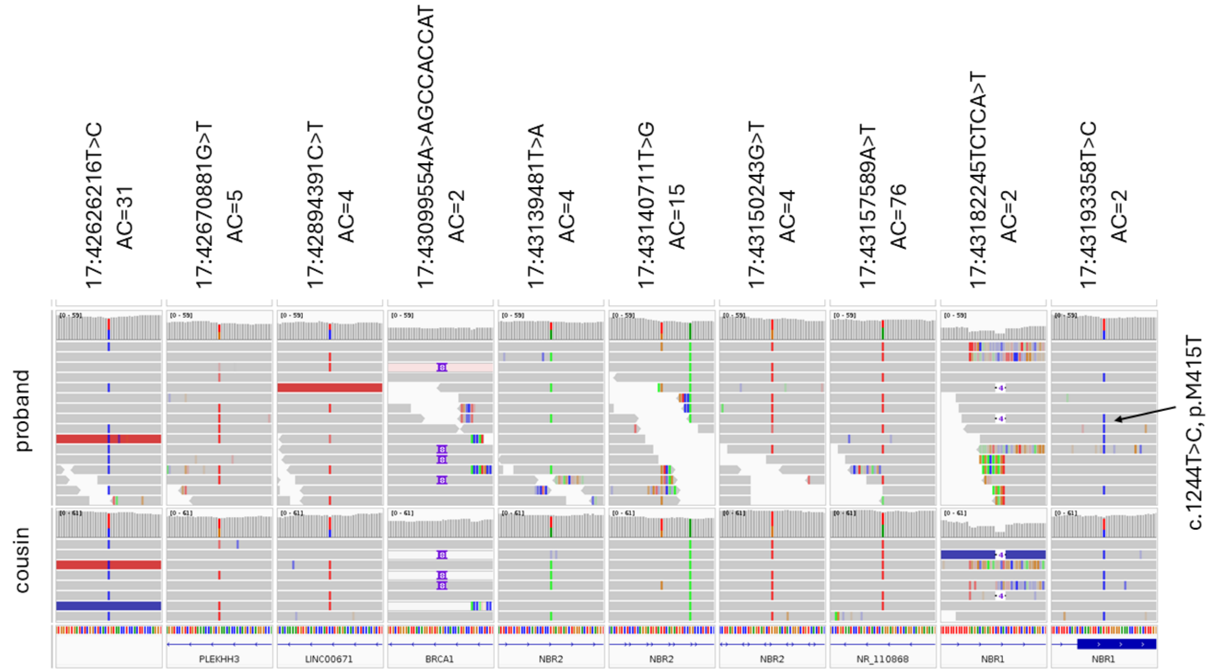

Figure S2: Read alignments supporting the closest 10 ultra-rare variants (8 SNVs, an 8bp duplication and a 4bp deletion) shared heterozygous between both affected individuals. cDNA and protein annotation for the single *NBR1* coding variant are based on NM_005899.5. AC, allele counts from aggregate data on 78,195 individuals from the 100k Genomes Project.

Table S1: Ultra rare variants likely *in cis* with deletion. Variants were extracted from the appropriate chunk of the 100kGP aggregate file due to an allele frequency of <0.1% and being shared heterozygous in both the proband and her cousin. Although, these variants are likely on the same haplotype as the deletion, phasing has not been formally confirmed and so using these variants as tagging variants should be applied with caution.

Genomic coordinate (GRCh38)	Distance from deletion	AN	AC
17:42626216T>C	473392	156390	31
17:42670881G>T	428727	156390	5
17:42894391C>T	205217	156390	4
17:43099554A>AGCCACCAT	54	156390	2
17:43099608-43099786del	NA	142816*	2
17:43139481T>A	39695	156390	4
17:43140711T>G	40925	156390	15
17:43150243G>T	50457	156390	4
17:43157589A>T	57803	156390	76
17:43182245TCTCA>T	82459	156382	2
17:43193358T>C†	93572	156389	2
17:43214013C>T	114227	156390	4
17:43333267T>C	233481	156390	16
17:43342505C>T	242719	156390	4
17:43354145G>A	254359	156390	4
17:43586428C>T	486642	156390	22
17:43697511T>C	597725	156390	5
17:43854244C>T	754458	156390	3
17:43969092G>A	869306	156389	12
17:43970126A>G	870340	156390	95
17:44013728G>A	913942	156390	13
17:44026122C>A	926336	156390	13
17:44056788G>T	957002	156390	11
17:44062206A>G	962420	156390	11
17:44095885G>A	996099	156390	112
17:44132865T>C	1033079	156384	2
17:44214721A>T	1114935	156388	90
17:44280026C>A	1180240	156390	2
17:44406864A>G	1307078	156390	119
17:44468763T>C	1368977	156390	3
17:44565442G>T	1465656	156390	50
17:44606265G>A	1506479	156390	116
17:44663309G>A	1563523	156388	116

*AN (effective allele number) is taken from the SVRare database[1] and so explains why the denominator is different from the smaller variants which were assessed using the AggV2 file (N=78,195 individuals). The first 10 variants that lie closest to the deletion are shown in Figure S2. †NM_005899.5(NBR1): c.1244T>C, p.Met415Thr is the only coding variant and thus may represent the best tagging SNV to search for this deletion in exon sequencing databases. AC, allele count.

Supplementary references

1. Yu, J., Szabo, A., Pagnamenta, A.T., Shalaby, A., Giacopuzzi, E., Taylor, J., Shears, D., Pontikos, N., Wright, G., Michaelides, M., et al. (2022). SVRare: discovering disease-causing structural variants in the 100K Genomes Project. medRxiv, 2021.2010.2015.21265069. 10.1101/2021.10.15.21265069.
